# Dissemination of *bla*_NDM-5_ and *mcr-8.1* in carbapenem-resistant *Klebsiella pneumoniae* and *Klebsiella quasipneumoniae* in an animal breeding area in Eastern China

**DOI:** 10.3389/fmicb.2022.1030490

**Published:** 2022-10-19

**Authors:** Chengxia Yang, Jingyi Han, Björn Berglund, Huiyun Zou, Congcong Gu, Ling Zhao, Chen Meng, Hui Zhang, Xianjun Ma, Xuewen Li

**Affiliations:** ^1^Department of Environment and Health, School of Public Health, Cheeloo College of Medicine, Shandong University, Jinan, Shandong, China; ^2^Department of Thoracic Surgery, Qilu Hospital of Shandong University, Jinan, Shandong, China; ^3^Department of Clinical and Experimental Medicine, Linköping University, Linköping, Sweden; ^4^Department of Blood Transfusion, Qilu Hospital of Shandong University, Jinan, Shandong, China

**Keywords:** carbapenem-resistant *Klebsiella* spp., animal breeding area, *bla*_NDM-5_, *mcr-8.1*, plasmid, clonal expansion

## Abstract

Animal farms have become one of the most important reservoirs of carbapenem-resistant *Klebsiella* spp. (CRK) owing to the wide usage of veterinary antibiotics. “One Health”-studies observing animals, the environment, and humans are necessary to understand the dissemination of CRK in animal breeding areas. Based on the concept of “One-Health,” 263 samples of animal feces, wastewater, well water, and human feces from 60 livestock and poultry farms in Shandong province, China were screened for CRK. Five carbapenem-resistant *Klebsiella pneumoniae* (CRKP) and three carbapenem-resistant *Klebsiella quasipneumoniae* (CRKQ) strains were isolated from animal feces, human feces, and well water. The eight strains were characterized by antimicrobial susceptibility testing, plasmid conjugation assays, whole-genome sequencing, and bioinformatics analysis. All strains carried the carbapenemase-encoding gene *bla*_NDM-5_, which was flanked by the same core genetic structure (IS*5*-*bla*_NDM-5_-*ble*_MBL_-*trpF*-*dsbD*-IS*26*-IS*Kox3*) and was located on highly related conjugative IncX3 plasmids. The colistin resistance gene *mcr-8.1* was carried by three CRKP and located on self-transmissible IncFII(K)/IncFIA(HI1) and IncFII(pKP91)/IncFIA(HI1) plasmids. The genetic context of *mcr-8.1* consisted of IS*903*-*orf*-*mcr-8.1-copR-baeS-dgkA*-*orf*-IS*903* in three strains. Single nucleotide polymorphism (SNP) analysis confirmed the clonal spread of CRKP carrying-*bla*_NDM-5_ and *mcr-8.1* between two human workers in the same chicken farm. Additionally, the SNP analysis showed clonal expansion of CRKP and CRKQ strains from well water in different farms, and the clonal CRKP was clonally related to isolates from animal farms and a wastewater treatment plant collected in other studies in the same province. These findings suggest that CRKP and CRKQ are capable of disseminating *via* horizontal gene transfer and clonal expansion and may pose a significant threat to public health unless preventative measures are taken.

## Introduction

The prevalence of antibiotic-resistant bacteria (ARB) and antibiotic-resistant genes (ARGs) has the potential to cause a global health crisis in the 21st century. Carbapenem-resistant *Klebsiella* spp. (CRK) are especially concerning owing to their resistance to carbapenems, one of the last resort antibiotics used against gram-negative bacteria, and ability to cause a variety of community- or hospital-acquired infections such as pneumonia, urinary tract infections, bloodstream infections, and septic shock. Carbapenem-resistant *Klebsiella pneumoniae* (CRKP), which is widely reported in Europe, Asia, Africa, and other regions (Navon-Venezia et al., 2017), is listed in the critical priority tier of pathogens and was recognized as the highest priority in new antibiotic development by the World Health Organization (WHO) in 2017 (Tacconelli et al., 2018). However, according to the China Antimicrobial Surveillance Network (CHINET),^1^ the prevalence of clinical meropenem resistance in *K. pneumoniae* has increased rapidly in China, from 2.9% in 2005 to 24.4% in 2021, posing a massive challenge to public health.

China is the largest consumer of veterinary antimicrobials, accounting for 45% of global use in 2017, and is expected to remain the largest consumer in 2030 (Tiseo et al., 2020). Thus, ARB and ARGs have been widely spread in animal farms, such as in animal feces (Tang et al., 2019), groundwater (Gu et al., 2022), aerosol (Yang et al., 2021), and animal foods (Wang et al., 2022). Although carbapenems are not approved for use on animal farms in China, the widespread use of other antibiotics such as fluoroquinolones, sulfonamides, and tetracyclines can co-select carbapenem-resistant bacteria (BIOHAZ, 2013). In China, CRK has been detected in animal farms, such as in animal feces and raw milk in dairy cattle farms in Jiangsu province (He et al., 2017), chicken cloaca and environment (sewage trenches, corridor floors, drooping boards, feeding troughs, and nipple drinkers) in broiler farms in Hebei province (Zhai et al., 2020), duck feces in Guangdong province (Ma et al., 2020), and pig anal swabs from a pig farm in Hunan province (Zhao et al., 2021b). However, most CRK studies on animal farms in China have two major shortcomings. First, the studies mainly focused on a single type of animal farms (He et al., 2017; Ma et al., 2020; Zhao et al., 2021b). Objectively understanding the true prevalence of CRK on different types of animal farms is difficult. Second, the studies are mostly limited to animals and the environment (Zhai et al., 2020; Gu et al., 2022), and the CRK transmission among animals, the environment, and humans is not entirely clear.

Thus, our study randomly selected 60 animal farms, including 20 chicken, 20 pig, and 20 bovine farms, in a typical region of the animal farming industry with a long breeding time and stable animal breeding patterns in Shandong province in July 2019. Based on the perspective of “One-Health,” animal feces, wastewater (excluding chicken farms), well water, and human feces were collected from each farm to screen for CRK. This study aimed to characterize CRK in terms of antimicrobial susceptibility, antibiotic resistance genes, virulence genes and multilocus sequence typing (MLST), investigate the prevalence of CRK in different types of farms in the same animal breeding area, and explore the transmission of CRK among animals, the environment, and humans.

## Materials and methods

### Sampling site and collection samples

**Figure 1:**
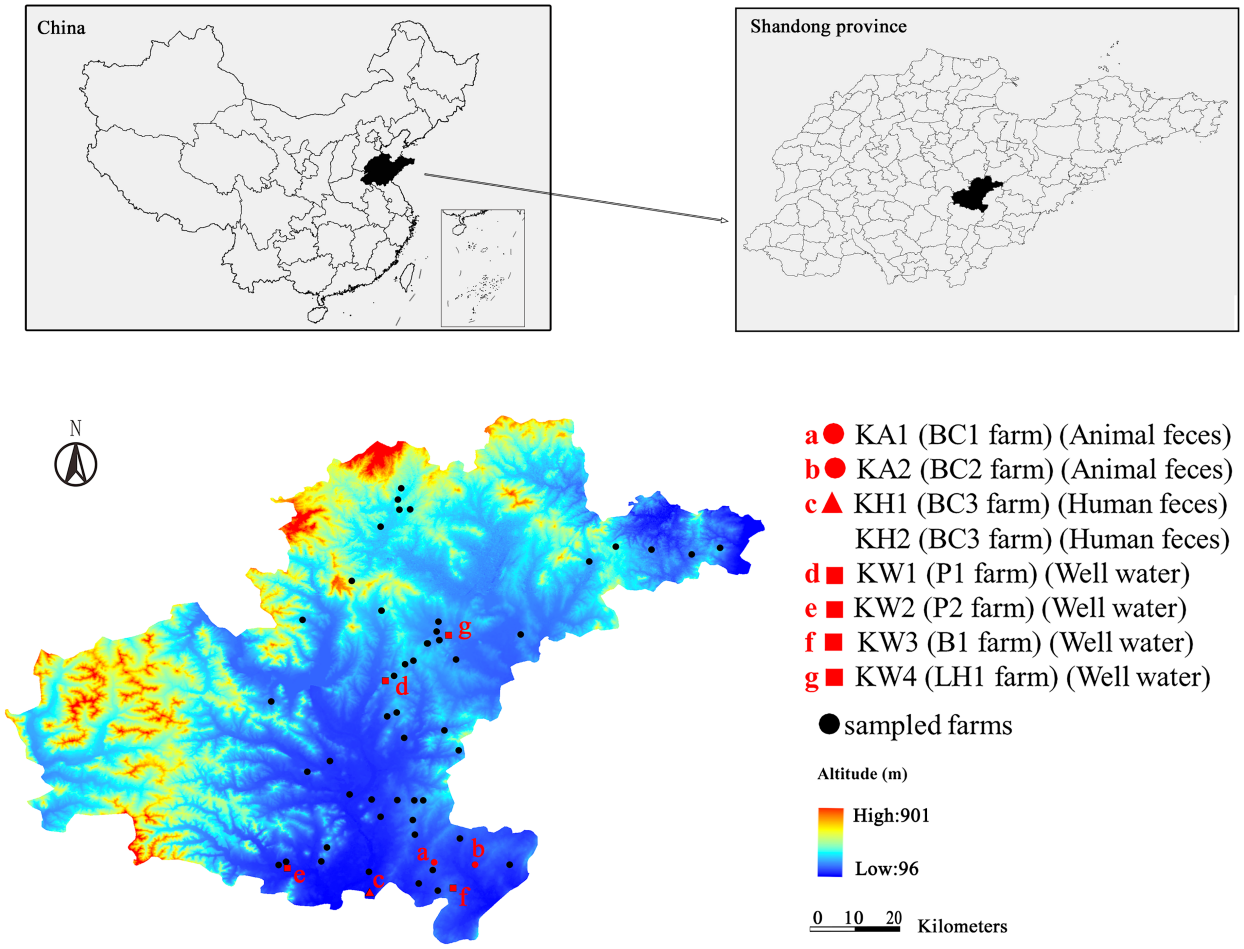
Map showing the location of the CRK-negative farms (denoted by black dots) and CRK-positive farms (denoted by red dots). The farms denoted “a” and “b” represent the farms in which CRK was isolated from animal feces. The red triangles (c) represent the farm in which CRK was isolated from human feces. Red squares (d, e, f, and g) represent the farms in which CRK was isolated from well water. BC1, BC2 and BC3 are broiler chicken farms; P1 and P2 are pig farms; B1 is a beef farm; LH1 is a laying hen farm.

The study site is in Eastern China, has a population of approximately 1,194,365, covers an area of 2,414 km^2^, and has a long breeding time and stable animal breeding patterns. The primary animal husbandry in this area consists of breeding chickens, pigs, and bovines. We selected 60 livestock and poultry farms (Figure 1), including pig farms (*n* = 20), chicken farms [(*n* = 20); laying hen farms (*n* = 11) and broiler farms (*n* = 9)], and bovine farms [(*n* = 20); dairy cattle farms (*n* = 13) and beef farms (*n* = 7)]. In July 2019, a total of 263 samples were collected, with 75 from chicken farms, 86 from pig farms, and 102 from bovine farms. The following samples types were collected: 66 animal feces, 37 wastewater, 107 worker feces, and 53 well water samples. The number and type of samples are summarized in Supplementary Table S1. Worker feces was collected using a Copan Liquid Amies Elution Swab (ESwab; Copan, Brescia, Italy). Well water (500 ml) from each farm well and wastewater (500 ml) from each farm sewage outlet (excluding chicken farms) were collected in sterile glass bottles. Approximately 30 g of each fresh animal fecal sample was collected in a sterile plastic bottle with a sterile spoon. All samples were temporarily placed in iceboxes (4–8°C) upon collection and transported to the laboratory at the end of the sampling day. The well water and wastewater samples were filtered through 0.45 μm sterile membrane filters (Millipore, Billerica, United States). After filtration, the membranes were placed in 5 ml of sterile brain heart infusion broth (BHI; Oxoid, Basingstoke, United Kingdom) with 20% glycerin. 2.0 g of each animal fecal sample was homogenized in 5 ml of sterile BHI broth (Oxoid, Basingstoke, United Kingdom) with 20% glycerin. All samples were stored at −80°C until cultivation.

### Isolation and identification for carbapenem-resistant *Klebsiella* spp.

For all samples, a pre-enrichment step in the BHI broth (Oxoid, Basingstoke, United Kingdom) was performed in a constant temperature shaker (Boxun, Shanghai, China) at 37°C overnight. Enriched solutions (50 μl) were evenly spread on MacConkey agar (Landbridge, Beijing, China) supplemented with 2 mg/l meropenem (Meilun, Dalian, China) to screen for carbapenem-resistant isolates. Colonies were selected based on colony morphotypes and repeatedly streaked on the MacConkey agar to obtain pure isolates (Zhao et al., 2021a). The isolates were tested for the presence of *bla*_NDM_, *bla*_KPC_, *bla*_OXA-48_, *bla*_VIM_ and *bla*_IMP_ carbapenemase genes using PCR, as previously described (Poirel et al., 2011). The PCR products were verified using Sanger sequencing (Biosune, Shanghai, China). Matrix-assisted laser desorption/ionization time-of-flight mass spectrometry (MALDI-TOF/MS; Bruker Daltonik GmbH, Bremen, Germany) was used to identify the carbapenem-resistant isolates. The CRK isolates were selected for further research.

### Antimicrobial susceptibility testing

The minimum inhibitory concentrations (MICs) of the 12 antibiotics were determined for CRK. 12 antibiotics were tested at a series of concentrations, and the maximum concentrations of amoxicillin/clavulanate, piperacillin/tazobactam, cefotaxime, ceftazidime, meropenem, imipenem, amikacin, ciprofloxacin, tetracycline, tigecycline, colistin and nitrofurantoin were 128/64 mg/l, 128/4 mg/l, 256 mg/l, 256 mg/l, 256 mg/l, 256 mg/l, 256 mg/l, 64 mg/l, 128 mg/l, 32 mg/l, 64 mg/l, 128 mg/l, respectively; the minimum concentrations were 0.0625 mg/l for 12 antibiotics. The MICs of tigecycline and colistin were determined using the broth microdilution method, and others were determined using the agar dilution method according to the Clinical Laboratory Standards Institute (CLSI). The results were interpreted according to the CLSI (document M100-S31), excluding tigecycline, for which MICs were interpreted following the ecological cut-off (ECOFF) of the European Committee on Antimicrobial Susceptibility Testing (EUCAST) SOP 10.2. *Escherichia coli* ATCC 25922 was used as the quality control strain. Multidrug resistance was defined as non-susceptibility to at least one agent in three or more antimicrobial categories (Magiorakos et al., 2012).

### The transmissibility of plasmids

Sodium azide-resistant *E. coli* J53 was used as the recipient strain, and CRK was used as the donor strain to evaluate the horizontal transferability of the plasmids mediating carbapenem resistance, colistin resistance, and tigecycline resistance by filter mating. The donor strain and recipient strain were mixed at a ratio of 2:1 and incubated at 37°C for 12 h. Transconjugants carrying carbapenemase gene, colistin resistance gene, and tigecycline resistance gene were selected by cultivation on LB agar supplemented with 100 mg/l sodium azide and 2 mg/l meropenem, 100 mg/l sodium azide and 2 mg/l colistin, 100 mg/l sodium azide and 4 mg/l tigecycline for 18 h at 37°C, respectively. Transconjugants carrying resistance genes were verified using PCR. Moreover, plasmids of tigecycline-resistant strain (KA2) were extracted using the M5 BAC/PAC large plasmid extraction kit (Mei5, Beijing, China) according to the manufacturer’s instructions. XL10-Gold chemically competent *E. coli* cells (Angyu, Shanghai, China) were used as the recipient strain to evaluate the transformation of plasmids that mediate tigecycline resistance using the heat shock method, as previously described (Hassan et al., 2022). Transformants were selected by cultivation on LB agar supplemented with 4 mg/l tigecycline for 18 h at 37°C.

### Whole-genome sequencing

All CRK were subjected to whole-genome sequencing on the Illumina NovaSeq 6,000-PE150 platform (Illumina, San Diego, United States) combined with the PacBio Sequel platform (Berry Genomics Co. Ltd.). Hybrid genome assembly with both short and long reads was performed using Unicycler v0.5.0 (Wick et al., 2017). The sequences were annotated using Prokka v1.12 (Seemann, 2014) and RAST v2.0 (Aziz et al., 2008). Antibiotic resistance genes, plasmid replicons, and MLST were identified at the Center for Genomic Epidemiology^2^ using ResFinder 4.1, PlasmidFinder 2.1, and MLST 2.0, respectively. Virulence genes were analyzed using the VFDB analyzer^3^ and capsule polysaccharide-based serotyping (K-type) was performed using the BIGSdb Klebsiella Pasteur MLST database.^4^ The Basic Local Alignment Search Tool (BLAST) at the National Center for Biotechnology Information (NCBI)^5^ was used to analyze the plasmid alignments. Alignment analysis of amino acid sequences of DNA gyrase (*gyrA* and *gyrB*) and topoisomerase IV (*parC* and *parE*) was performed using Clustal Omega (Sievers et al., 2011). Genetic relatedness of the isolates was determined by single nucleotide polymorphism (SNP) analysis using CSI Phylogeny 1.4 at the Center for Genomic Epidemiology (Kaas et al., 2014). A distance of ≤15 SNPs was defined as clonally related strains (Schürch et al., 2018). Visualization of the genome comparison of the plasmids harboring *bla*_NDM-5_ and *mcr-8.1* was performed using BLAST Ring Image Generator (BRIG; Alikhan et al., 2011). All genome sequences in this study have been deposited in NCBI Genome database under BioProject PRJNA834640. The *bla*_NDM-5_-carrying plasmids of strains KA1, KA2, KH1, KH2, KW1, KW2, KW3, and KW4 have been deposited into GenBank under accession nos. CP102896.1, CP102875.1, CP102881.1, CP102887.1, CP102900.1, CP102890.1, CP102892.1, and CP102905.1, respectively. The *mcr-8.1*-carrying plasmids of strains KA2, KH1 and KH2 have been deposited into GenBank under accession nos. CP102872.1, CP102880.1, and CP102886.1, respectively.

## Results

### Prevalence of carbapenem-resistant *Klebsiella* spp.

Eight CRK were isolated from 263 samples, five of which were *K. pneumoniae* and three were *K. quasipneumoniae*. Five CRK (KA1, KA2, KH1, KH2, and KW4) were isolated from chicken farms (6.7%, 5/75), two (KW1 and KW2) from pig farms (2.3%, 2/86) and one (KW3) from bovine farms (0.98%, 1/102). KH1 and KH2 were isolated from the same chicken farm, whereas the other strains were obtained from different farms. In terms of sample types, four CRK (KW1-KW4) were isolated from well water, two (KA1 and KA2) from animal feces, two (KH1 and KH2) from human feces, and no CRK was isolated from wastewater (Supplementary Table S1).

### Phenotypic and genotypic antibiotic resistance

Antibiotic susceptibility testing showed that all CRK were multidrug-resistant and resistant to amoxicillin-clavulanic acid, piperacillin/tazobactam, cefotaxime, ceftazidime, meropenem, imipenem, and tetracycline. Most isolates were resistant to ciprofloxacin (62.5%), whereas resistance to colistin (50%), amikacin (37.5%), nitrofurantoin (37.5%), and tigecycline (12.5%) was observed to a lesser degree (Table 1). Eight CRK harbored the carbapenemase gene *bla*_NDM-5_, accounting for high-level resistance to meropenem (≥128 mg/l) and imipenem (≥64 mg/l). In addition to the carbapenemase gene, CKR carried various classes of resistance genes and the most prevalent ARGs (prevalence ≥50%), including *oqxAB* (*n* = 8), *fosA* (*n* = 8), *tet* (A) (*n* = 6), *aadA2* (*n* = 5), *floR* (*n* = 5), *qnrS1* (*n* = 4), and *aac(6′)-Ib-cr* (*n* = 4; Supplementary Table S2). Three strains (KA2, KH1, and KH2) carried the colistin resistance *mcr-8.1* and *armA* genes which encode a 16S rRNA-methylase, accounting for high-grade aminoglycoside resistance. KH1 and KH2 were resistant to all tested antibiotics except tigecycline, and KA2 was resistant to all tested antibiotics, including tigecycline. However, no known tigecycline resistance genes were not found on KA2 strain. Plasmid conjugation assay and transformation assay found that the plasmids pKA2-2, pKA2-3-mcr8.1, pKA2-5, and pKA2-6-NDM5 could be successfully transferred from KA2 to the recipient strain *E. coli* J53, while the transconjugants and transformants were remain sensitive to tigecycline, indicating a novel or more complex resistant mechanism in this strain.

**Table 1:** Antimicrobial susceptibility profiles of carbapenem-resistant *Klebsiella* spp. strains from animal farms in Shandong, China.

Isolate	Species	Farm^a^	Source	Minimal inhibitory concentration (mg/L)											
				AMC	TZP	CTX	CAZ	MEM	IPM	AMK	CIP	TE	TGC	CL	F
KA1	*K. quasipneumoniae*	BC1	Animal feces	**32/16**	**>128/4**	**>256**	**>256**	**128**	**64**	4	**8**	**128**	1	1	32
KA2	*K. pneumoniae*	BC2	Animal feces	**32/16**	**>128/4**	**>256**	**>256**	**128**	**64**	**>256**	**8**	**>128**	**8**	**>64**	**>128**
KH1	*K. pneumoniae*	BC3	Human feces	**32/16**	**>128/4**	**>256**	**>256**	**256**	**256**	**>256**	**>64**	**128**	1	**4**	**>128**
KH2	*K. pneumoniae*	BC3	Human feces	**32/16**	**>128/4**	**>256**	**>256**	**128**	**128**	**>256**	**>64**	**128**	1	**4**	**>128**
KW1	*K. quasipneumoniae*	P1	Well water	**32/16**	**>128/4**	**>256**	**>256**	**128**	**128**	16	**4**	**128**	1	**4**	32
KW2	*K. pneumoniae*	P2	Well water	**32/16**	**>128/4**	**>256**	**>256**	**128**	**64**	2	0.25	**128**	0.5	1	64
KW3	*K. pneumoniae*	B1	Well water	**64/32**	**>128/4**	**>256**	**>256**	**128**	**64**	2	0.25	**64**	0.25	2	64
KW4	*K. quasipneumoniae*	LH1	Well water	**64/32**	**>128/4**	**>256**	**>256**	**128**	**64**	2	0.5	**128**	0.25	1	16
ATCC 25922	*E. coli*			8/4	1/4	0.06	0.25	0.03	0.5	2	0.03	1	0.5	1	4

a BC1, BC2 and BC3 are broiler chicken farms; P1 and P2 are pig farms; B1 is a beef farm; LH1 is a laying hen farm.

AMC, amoxicillin/clavulanate; TZP, piperacillin/tazobactam; CTX, cefotaxime; CAZ, ceftazidime; MEM, meropenem; IPM, imipenem; AMK, amikacin; CIP, ciprofloxacin; TE, tetracycline; TGC, tigecycline; CL, colistin; F, nitrofurantoin. Resistance is indicated in bold, intermediate resistance is indicated by underlined.

As the strains showed a high resistance rate (62.5%, 5/8) to ciprofloxacin, the resistance mechanisms of ciprofloxacin were analyzed. Comparison of the amino acid sequence of DNA gyrase (*gyrA* and *gyrB*) and topoisomerase IV (*parC* and *parE*) between the CRK isolated in this study and the reference sequence in the NCBI database showed that the amino acid substitutions of quinolone resistance-determining regions (QRDRs) were observed at position 83 (Ser → IIe) of *gyrA* and position 80 (Ser → IIe) of *parC* in KA2, KH1, and KH2 (Supplementary Table S3). Three strains were resistant to ciprofloxacin, two of which had high-level ciprofloxacin resistance (MICs >64 mg/l). Several studies have confirmed that amino acid co-substitution in the QRDRs of *gyrA* and *parC* in *Klebsiella* spp. were associated with high-level resistance to ciprofloxacin (Minarini and Darini, 2012; Kareem et al., 2021; Zhan et al., 2021), which is consistent with our results. Additionally, all CRK, including ciprofloxacin-resistant strains, carried three or more fluoroquinolone resistance genes, including *oqxAB* (100%, 8/8), *qnrS* (50%, 4/8), *qnrB* (50%, 4/8), and *aac(6′)-Ib-cr* (50%, 4/8; Supplementary Table S3). Even in the absence of QRDRs mutations, one strain carrying the fluoroquinolone resistance genes *qnrB2*, *aac(6′)-Ib-cr,* and *oqxAB*, and another strain carrying *qnrS1* and *oqxAB* showed non-susceptibility to ciprofloxacin. The *qnr* genes encode proteins belonging to the pentapeptide repeat protein family that play a role in binding and protecting topoisomerase IV and DNA gyrase from repression by ciprofloxacin (Kim et al., 2009). The *aac(6′)-Ib-cr* gene encodes a variant aminoglycoside acetyltransferase that reduces the activity of ciprofloxacin by N-acetylation at the amino nitrogen on its piperazinyl substituent (Robicsek et al., 2006). Mirzaii et al. (2018), Zhan et al. (2021), and Reo et al. (2022) reported that *Klebsiella* spp. strains carrying fluoroquinolone resistance genes, but without mutations in the QRDRs, were resistant to ciprofloxacin. These findings indicate that fluoroquinolone resistance genes can mediate ciprofloxacin resistance in *Klebsiella* spp.

### Virulence genes of carbapenem-resistant *Klebsiella* spp.

In this study, all strains possessed the type 1 fimbrial gene cluster *fimABCDEFGHI*, type 3 fimbrial gene cluster *mrkABCDFHIJ*, enterobactin-encoding genes *entABCDEFS* and its transport protein-encoding genes *fepABCDG*, and salmochelin-encoding gene *iroE*, but carried no genes related to hypervirulent *Klebsiella*, such as *iroB, iucA, rmpA and rmpA2* (Thomas et al., 2018; Supplementary Table S2).

### Genotypic characterization of carbapenem-resistant *Klebsiella* spp.

The silico analysis of the general molecular characteristics of the isolates are summarized in Supplementary Table S2. The eight strains belonged to four different sequence types (ST): ST526 (KA1, KW1, and KW4), ST37 (KH1 and KH2), ST766 (KW2 and KW3), and ST495 (KA2). Three types of capsular serotypes KL22KL37-*wzi*385, KL28-*wzi*84, and KL15KL17KL51KL52-*wzi*50 were identified in KH1 and KH2, KW1 and KW4, and KW2 and KW3, respectively, and KA1 and KA2 had poor match confidence against any known serotype and were determined to be non-typeable (Supplementary Table S2). The genome of each strain possessed a circular chromosome with sizes ranging from 5,225,745 bp to 5,320,447 bp. Furthermore, the genomes of KA1, KA2, KH1, KH2, KW1, KW2, KW3, and KW4 contained four, seven, five, five, four, one, one, and five circular plasmids, respectively. As shown in Supplementary Table S2, various classes of resistance genes were detected on the chromosomes and plasmids of the strains. Incompatible IncX3 type plasmids (45,122–52,085 bp) carrying *bla*_NDM-5_ were found on all strains. A hybrid incompatible IncFII(pKP91)/IncFIA(HI1) plasmid carrying *mcr-8.1* was detected on KA2, and IncFII(K)/IncFIA(HI1) plasmids carrying *mcr-8.1* were detected on KH1 and KH2.

### Characterization and comparative genomics of plasmids carrying *bla*_NDM-5_ and *mcr-8.1*

**Figure 2:**
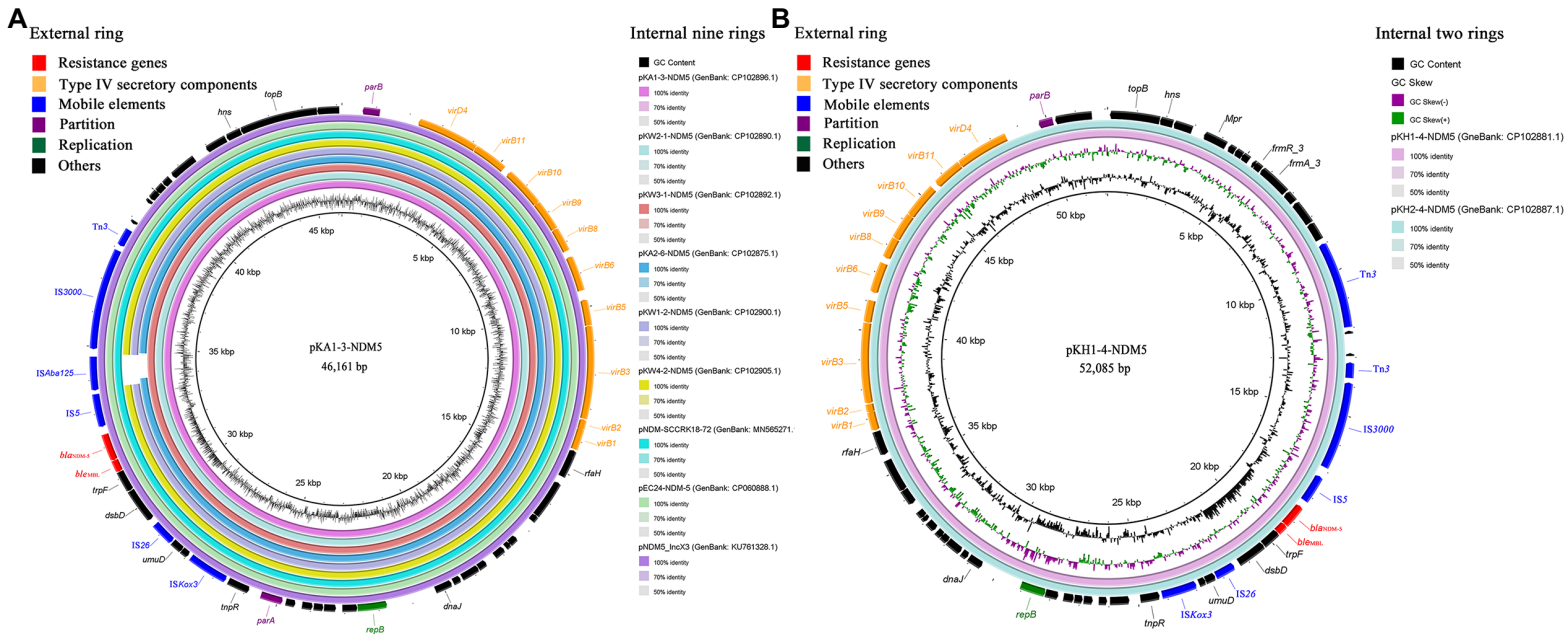
The blast and annotation of *bla*_NDM-5_-carrying IncX3 plasmid genomes (constructed by BRIG). Genes are color-coded depending on functional annotations. **(A)** The nine internal rings represent different *bla*_NDM-5_-carrying IncX3 plasmids from this study and GenBank database. The external ring represents the annotation of plasmid pKA1-3-NDM5. **(B)** The two internal rings represent plasmid pKH1-4-NDM5 and pKH2-4-NDM5. The external ring represents the annotation of plasmid pKH1-4-NDM5.

*bla*_NDM-5_-carrying IncX3 plasmids were successfully transferred from all strains to the recipient strain *E. coli* J53 *via* conjugation assays. *bla*_NDM-5_ was located on the 46,161 bp IncX3 plasmid among strains KA1, KW2, and KW3, designated pKA1-3-NDM5, pKW2-1-NDM5, and pKW3-1-NDM5, respectively. Plasmid sequence comparison showed that the three IncX3 plasmids shared 100% nucleotide identity and 100% coverage with each other, and were identical to several other IncX3 plasmids found in *Enterobacteriaceae* isolates in China, such as the plasmid pNDM5_IncX3 (GenBank: KU761328.1) from *K. pneumoniae* isolated from a patient in Suzhou city, pNDM-SCCRK18-72 (GenBank: MN565271.1) from an *E. coli* isolated from chicken in Sichuan province, and pEC24-NDM-5 (GenBank: CP060888.1) from an *E. coli* isolated from a patient in Zhejiang province. *bla*_NDM-5_ was flanked by the same genetic structure as that of Tn*3*-IS*3000*-IS*Aba125*-IS*5*-*bla*_NDM-5_-*ble*_MBL_-*trpF*-*dsbD*-IS*26*-IS*Kox3* (Figure 2A). In strains KA2, KW1, and KW4, *bla*_NDM-5_ was located on 45,244, 45,122 and 45,122 bp IncX3 plasmids, designated pKA2-6-NDM5, pKW1-2-NDM5, and pKW4-2-NDM5, respectively. The three IncX3 plasmids showed 97% coverage and 99.99% nucleotide identity with pKA1-3-NDM5. Tn*3*, IS*3000*, and IS*5* were located upstream of *bla*_NDM-5_, whereas *ble*_MBL_, *trpF*, *dsbD,* IS*26*, and IS*Kox3* were located downstream (Figure 2A). KH1 and KH2 contained a 52,085 bp IncX3 plasmid carrying *bla*_NDM-5_, which shared 99.99% nucleotide identity and 97% coverage with pKA1-3-NDM5. The environment of *bla*_NDM-5_ was same with strains KA2, KW1, and KW4 (Figure 2B). Although the size and structure of IncX3 plasmids carrying *bla*_NDM-5_ differed slightly among strains, all IncX3 plasmids contained a highly conserved region around *bla*_NDM-5_, consisting of IS*5*-*bla*_NDM-5_-*ble*_MBL_-*trpF*-*dsbD*-IS*26*-IS*Kox3*.

**Figure 3:**
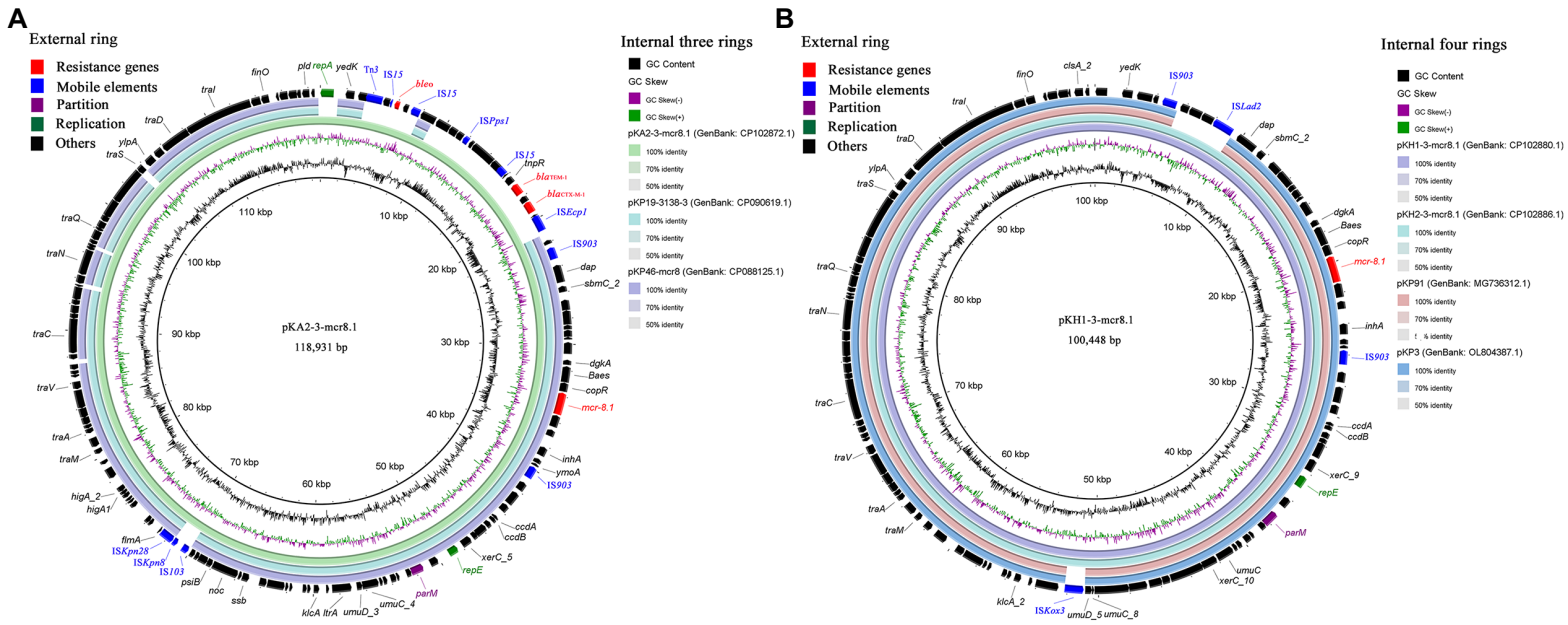
The blast and annotation of *mcr-8.1*-carrying plasmid genomes (constructed by BRIG). Genes are color-coded depending on functional annotations. **(A)** The three internal rings represent plasmids pKA2-3-mcr8.1, pKP19-3,138-3 (GenBank: CP090619.1) and pKP46-mcr8 (GenBank: CP088125.1). The external ring represents the annotation of plasmid pKA2-3-mcr8.1. **(B)** The four internal rings represent different *mcr-8.1*-carrying plasmids from this study and GenBank database. The external ring represents the annotation of plasmid pKH1-3-mcr8.1.

The *mcr-8.1* harboring plasmids were successfully transferred into the recipient strain *E. coli* J53 from three CRKP *via* conjugation assays. In strain KA2, *mcr-8.1* was located on a 118,931 bp plasmid, designated pKA2-3-mcr8.1, which contained IncFII(pKP91) and IncFIA(HI1) replicons. In addition to *mcr-8.1*, resistance genes *bla*_TEM-1B_, *bla*_CTX-M-1_, and *ble_O_* were identified on the plasmid. The plasmid BLAST query in the GenBank database showed that the plasmid pKA2-3-mcr8.1 exhibited the highest degree of sequence homology with plasmids pKP19-3,138-3 (GenBank: CP090619.1) from *K. pneumoniae* and pKP46-mcr8 (GenBank: CP088125.1) from *K. pneumoniae*, with over 99% identity and 81% coverage (Figure 3A). In strains KH1 and KH2, *mcr-8.1* was located on 100,448 bp plasmids, designated pKH1-3-mcr8.1 and pKH2-3-mcr8.1, both of which harbor IncFII(K) and IncFIA(HI1) replicons. The two plasmids shared over 99% nucleotide identity and 94% coverage with the plasmids pKP3 (GenBank: OL804387.1) and pKP91 (GenBank: MG736312.1), which were isolated from chicken anal swab and pig feces, respectively, in Shandong province, China (Figure 3B), indicating that this type of plasmid may have been widely spread in this region. The genetic context of *mcr-8.1* in the isolates was similar and was composed of *IS903-orf-mcr-8.1-copR-baeS-dgkA-orf-IS903*. *mcr-8.1* was flanked by the complete insertion sequence IS*903*, which may facilitate the transmission of *mcr-8.1* among animals, the environment, and humans in this animal breeding area. Additionally, the *mcr-8.1*-carrying IncFII(pKP91)/IncFIA(HI1) and IncFII(K)/IncFIA(HI1) plasmids carried two genes coding the response regulator transcription factor *CopR* and HAMP domain-containing histidine kinase *Baes*. The combination of these two protein families usually constitutes a two-component system involved in colistin resistance in *Enterobacteriaceae*.

### Genetic relationship analysis

**Figure 4:**
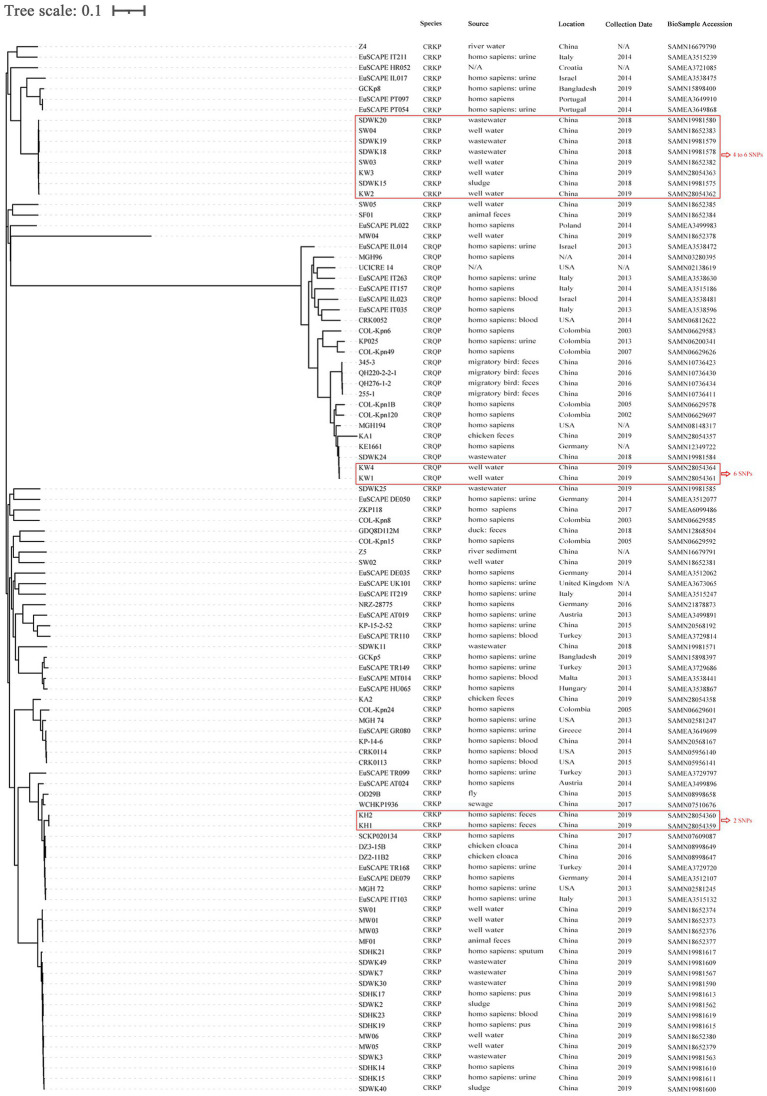
SNP-based phylogenetic tree of 8 carbapenem-resistant *Klebsiella* spp. isolated from animal breeding area in this study, and another 72 carbapenem-resistant *Klebsiella pneumoniae* (CRKP) and 20 carbapenem-resistant *Klebsiella quasipneumoniae* (CRKQ) in NCBI database. The sample identifier, location, collection date and source were indicated for each isolate. The number of SNPs denoted in red outside the rectangles indicate the number of SNPs differing between the isolates. *K. pneumoniae* MGH 78578 (GenBank: NC_009648.1) genome sequence was used as reference for construction of the phylogenetic trees based on SNP differences. N/A, not available.

SNP-based phylogenetic analysis revealed that the eight CRK were grouped into four clusters. The first cluster included KW2 and KW3, isolated from the well water of farms P2 and B1, respectively, with four SNPs differences. The second cluster consisted of KA1, KW1, and KW4, among which KW1 and KW4 differed by only six SNPs and were isolated from the well water of farms P1 and LB1. The third cluster included only KA2. The fourth cluster included KH1 and KH2 isolated from two human fecal samples in farm BC3, with two SNPs differences (Figure 4; Supplementary Table S4).

Moreover, the genetic relationships between these strains, and 72 CRKP and 20 carbapenem-resistant *Klebsiella quasipneumoniae* (CRKQ) stains from the GenBank database were characterized (Figure 4). The sample identifier (ID), location, collection date, and source were indicated for each strain. The results suggest that CRKP and CRKQ were wide spread among humans, animals, and different environmental media (well water, sewage, sludge, river water, and river sediment) in multiple countries, and humans were the most common hosts of CRKP and CRKQ. KW2 and KW3 were clonally related to four CRKP from sludge and sewage in a wastewater treatment plant (WWTT), including *K. pneumoniae* strains SDWK15, SDWK18, SDWK19, and SDWK20, and two CRKP (*K. pneumoniae* strains SW03 and SW04) from well water in two animal breeding areas (differed by 4–6 SNPs). KW1 and KW4 were similar to *K. quasipneumoniae* strain KE1661 from a human rectal swab in Germany (differed by 89 and 87 SNPs, respectively) and *K. quasipneumoniae* strain SDWK24 from the abovementioned WWTT (differed by 91 and 91 SNPs, respectively; Supplementary Table S4). The WWTT and animal breeding areas are in different counties of Shandong province, more than 200 km and 150 km from this study site, respectively.

## Discussion

As animal farms have become one of the most important reservoirs of CRK, it is important to investigate the prevalence and dissemination of CRK in animal breeding areas. In this study, a total of eight *bla*_NDM-5_-carrying CRK were isolated from animal feces, well water, and worker feces from seven animal farms. Four CRK were isolated from animal feces and human feces in chicken farms, whereas no CRK was isolated from animal feces and human feces in pig and bovine farms. A possible explanation for this may be the use of antibiotics on the farms. Antibiotics are usually used to prevent and treat diseases in larger quantities and at higher frequencies in chicken farms than in pig and bovine farms, because of the higher breeding density and shorter production cycles of chicken farms, especially broiler farms (Zhao et al., 2010). Several studies have shown results consistent with our research. For example, Gu et al. observed that the carbapenem-resistant *Enterobacteriaceae* (CRE) detection rate in animal fecal samples was higher in chicken farms compared to bovine and pig farms in northern China (Gu et al., 2022). Qian et al. found that the diversity and abundance of ARGs were higher in chicken feces than in bovine feces sampled from the Shaanxi province in China (Qian et al., 2018). In this study, four CRK were isolated from well water in chicken, pig, and bovine farms, suggesting that groundwater has been contaminated by CRK in this animal breeding area and groundwater may have become a potential CRK reservoir. In addition to CRK, other carbapenem-resistant *Enterobacteriaceae* species have been detected in well water in animal breeding areas in China. Sun et al. isolated one *bla*_KPC-2_-carrying *Raoultella ornithinolytica* from well water in a pig breeding area in Shandong province (Sun et al., 2017). Zou et al. detected one *bla*_NDM-1_-carrying *Raoultella ornithinolytica* in well water in Weifang city of Shandong province (Zou et al., 2020). Gu et al. conducted a large-scale study in animal breeding areas of six countries in Inner Mongolia and Shandong province, and detected several CRE from well water, including *K. pneumoniae*, *Enterobacter* spp., *Citrobacter sedlakii*, and *K. michiganensis* (Gu et al., 2022). The results indicate that the groundwater in animal breeding areas is vulnerable to CRE contamination, and it is necessary to strengthen the groundwater monitoring and control of CRE in breeding areas.

Since the IncX3 plasmid pNDM-MGR194 carrying *bla*_NDM-5_ was first identified in *K. pneumoniae* from a patient in India (Krishnaraju et al., 2015), pNDM-MGR194-like plasmids have been found in *Enterobacteriaceae* worldwide (Alexander et al., 2015; Tian et al., 2020; Chakraborty et al., 2021; Costa et al., 2021). Additionally, previous studies have shown a high capacity of the IncX3 plasmid to mediate the dissemination of *bla*_NDM-5_ in *Enterobacteriaceae* in various environments. Tian et al. reported that the horizontal gene transfer (HGT) of *bla*_NDM-5_ among different *Enterobacteriaceae* species was mainly mediated by IncX3 plasmids in a pediatric hospital in Shanghai (Tian et al., 2020). Gu et al. confirmed that *bla*_NDM-5_-carrying IncX3 plasmids are widely disseminated among different *Enterobacteriaceae* genera among animals and the environment in animal breeding areas in Inner Mongolia and Shandong province (Gu et al., 2022). Zhao et al. reported that *bla*_NDM-5_ could disseminate among humans and the environment *via* IncX3 plasmids in an intensive vegetable cultivation area in Shandong province (Zhao et al., 2021a). In this study, *bla*_NDM-5_ was located on highly related conjugative IncX3 plasmids among all strains and flanked by the same core genetic structure: IS*5*-*bla*_NDM-5_-*ble*_MBL_-*trpF*-*dsbD*-IS*26*-IS*Kox3*, which confirms that the IncX3 plasmid may serve as a major vehicle for the *bla*_NDM-5_ dissemination among animals, the environment, and humans in this animal breeding area.

KH1 and KH2 from two fecal samples from workers at farm BC3 and KA2 from animal feces at farm BC2 carried the colistin resistance gene *mcr-8.1* and were resistant to colistin. Colistin is effective against most gram-negative bacteria and is considered a last-resort antibiotic for treating serious infections caused by multidrug-resistant bacteria, particularly CRE (Li et al., 2006; Petrosillo et al., 2019). However, with the emergence and prevalence of CRE, extensive use of colistin has led to the emergence of colistin resistance. In 2016, Liu et al. reported the plasmid-mediated colistin resistance gene *mcr-1* in *Enterobacteriaceae* isolated from animals and humans in China (Liu et al., 2016). In the past 5 years, *mcr* gene variants (*mcr-1* to *mcr-10*) have been identified in different bacteria isolated from animals, the environment, and humans worldwide (Hussein et al., 2021; Portes et al., 2022). Since Wang et al. reported that *mcr-8.1* is located on a conjugative IncFII-type plasmid in *K. pneumoniae* in 2018 (Wang et al., 2018), *K. pneumoniae* has become one of the main hosts of *mcr-8.1* in animals and humans. Plasmids are considered to play an important role in the dissemination of *mcr-8.1* genes, because the majority of *mcr-8.1* carriers are on plasmids. *Mcr-8.1* has been reported to be located on various plasmids replicon types, especially IncFII, IncFIA, IncFIB, IncQ, IncR, and IncA/C replicon plasmids in *Enterobacteriaceae* (Hadjadj et al., 2019; Farzana et al., 2020; Wu et al., 2020). In this study, *mcr-8.1* was located on the hybrid incompatible plasmid groups IncFII(pKP91)/IncFIA(HI1) and IncFII(K)/IncFIA(HI1), supporting the notion that plasmids are the main factor in disseminating *mcr-8.1*.

Clonal spread of colistin-resistant CRKP has been reported most frequently in hospitals (Battikh et al., 2016; Jonathan et al., 2017; Rocha et al., 2022). However, several studies have reported the clonal spread of colistin-resistant CRKP in animal farms. For example, Zhai et al. and Sun et al. reported the clonal spread of colistin-resistant CRKP among chickens and the environment in a poultry farm in Hebei province, China and among chickens in a chicken farm in Shandong province, China, respectively (Sun et al., 2020; Zhai et al., 2020). In this study, SNP analysis confirmed the clonal spread of ST37 *K. pneumoniae* carrying *bla*_NDM-5_ and *mcr-8.1* between human workers in the same chicken farm. However, because the types of samples were limited, the route of dissemination could not be determined in this study, and further research should clarify the route of clonal spread and provide effective control strategies for the dissemination of colistin-resistant CRKP in animal farms. Additionally, two pairs of clonally related CRKP and CRKQ were isolated from well water in four different farms located at a distance of more than 20 km, revealing that clonal spread also has significant implications for the dissemination of *bla*_NDM-5_ in the animal breeding area. Unexpectedly, the pair of clonal CRKP was clonally related to six strains from animal farms and a WWTT collected in other studies in the same province, suggesting that *bla*_NDM-5_-carrying *K. pneumoniae* are capable of disseminating within or between farms in the same county, even in different counties within a province, *via* clonal expansion. Although the exact route of long-distance transmission of CRKP is uncertain, population movement and travel (Schwartz and Morris, 2018), migration of birds (Wang et al., 2017), and the live poultry trade (Wang et al., 2019) may promote the cross-regional transmission of CRKP.

## Conclusion

In this study, we investigated the prevalence of CRK by randomly selecting 20 chicken, 20 pig, and 20 bovine farms from a typical region of the animal farming industry in China. Based on the “One-Health” concept, the transmission of CRK among animals, the environment and humans were discussed. All CRK carried *bla*_NDM-5_, which was located on highly related conjugative IncX3 plasmids. *Mcr-8.1* was carried by three CRK and located on self-transmissible IncFII(K)/ IncFIA(HI1) and IncFII(pKP91)/IncFIA(HI1) plasmids. Two pairs of clonal CRK from well water and one pair of clonal colistin-resistant CRK from human workers were identified. Thus, both HGT *via* plasmids and clonal expansion play a significant role in disseminating *bla*_NDM-5_ and *mcr-8.1* among animals, the environment, and humans in the animal breeding area. The results highlight that systematical monitoring and large-scale investigation of the prevalence and spread of CRK with a “One Health”-perspective in animal breeding areas in Eastern China are urgently needed to enable effective measures for controlling the further spread of CRK.

## Data availability statement

The datasets presented in this study can be found in online repositories. The names of the repository/repositories and accession number(s) can be found in the article/Supplementary material.

## Ethics statement

Ethical approval to conduct this study was granted by the Ethics Committee for Shandong University [Ethics permission number: 20191202]. The patients/participants provided their written informed consent to participate in this study. Ethical review and approval was not required for the animal study because We only collected animal feces from the animal farms and did not contact animals.

## Author contributions

CY: sampling, experiments conducting, data formal analysis, and writing—original draft preparation. BB and JH: writing—reviewing and editing. HZo, CG, LZ, CM, and HZh sampling. XM and XL: conceptualization, supervision, and writing-reviewing. All authors contributed to the article and approved the submitted version.

## Funding

This work was supported by the National Key Research and Development Program of China [2020YFC1806904] and the National Natural Science Foundation of China (grant nos. 8197120700 and 41771499).

## Conflict of interest

The authors declare that the research was conducted in the absence of any commercial or financial relationships that could be construed as a potential conflict of interest.

## Publisher’s note

All claims expressed in this article are solely those of the authors and do not necessarily represent those of their affiliated organizations, or those of the publisher, the editors and the reviewers. Any product that may be evaluated in this article, or claim that may be made by its manufacturer, is not guaranteed or endorsed by the publisher.

## Supplementary material

The Supplementary material for this article can be found online at: https://www.frontiersin.org/articles/10.3389/fmicb.2022.1030490/full#supplementary-material

## Footnotes

^1^ http://www.chinets.com/

^2^ http://www.genomicepidemiology.org/services/

^3^ http://www.mgc.ac.cn/cgi-bin/VFs/v5/main.cgi

^4^ https://bigsdb.pasteur.fr/klebsiella/

^5^ https://blast.ncbi.nlm.nih.gov/Blast.cgi
